# Comparing keystone and Limberg flaps for pilonidal sinus: Does flap choice influence patient satisfaction?

**DOI:** 10.1007/s10151-025-03283-4

**Published:** 2026-05-07

**Authors:** Mohamed Wael, Abdelhamid Ghazal, Ahmed Ismail Ibrahim, Mostafa Refaie Elkeleny, Ahmed Abdelfattah Sabry, Mostafa Seif

**Affiliations:** 1 Alexandria Main University Hospital, Alexandria, Egypt; 2 Alexandria University, Alexandria, Egypt

**Keywords:** Pilonidal sinus, Patient satisfaction, Keystone (KSF), Limberg flap (LF)

## Abstract

**Background:**

Pilonidal sinus disease (PSD) is a chronic condition that mainly affects young adults impairing the quality of life. Among several surgical options, flap reconstruction offers faster healing and lower recurrence. This randomized comparative study evaluated the keystone perforator island flap (KSF) versus the Limberg flap (LF) in PSD repair, focusing primarily on patient satisfaction depending on the flap type, alongside perioperative outcomes following PS excision as secondary outcomes.

**Methods:**

A total of 50 patients with simple PSD were randomized equally to undergo KSF or LF reconstruction after sinus excision. Operative data, complications, and recovery metrics were recorded. Patient satisfaction, the primary endpoint, was assessed at 6 months using a validated 5-point scale. Statistical analysis employed the *t*-test, chi-squared test, and multivariate linear regression to identify independent predictors of satisfaction (*p* < 0.05 significant).

**Results:**

The KSF group showed shorter operative time (54.9 ± 3.7 versus 73.4 ± 7.9 min, *p* < 0.001), faster wound healing (13.5 ± 3.1 versus 17.1 ± 3.5 days, *p* < 0.001), and earlier return to activity (6.8 ± 0.8 versus 8.5 ± 0.8 days, *p* < 0.001). Complications were lower (24% versus 56%, *p* = 0.02). Satisfaction was significantly higher with KSF (96% versus 72%, *p* = 0.046). Regression analysis identified wound-healing time (*p* = 0.008) and return to activity (*p* < 0.001) as the independent predictors of satisfaction.

**Conclusion:**

The KSF provides faster recovery, better comfort, and higher patient satisfaction than the LF. Functional recovery parameters, rather than flap type alone, are the strongest determinants of postoperative satisfaction.

## Introduction

Pilonidal sinus disease (PSD) is a chronic inflammatory condition that primarily affects young male individuals in the sacrococcygeal region, leading to recurrent infection, pain, and impaired quality of life [1–4]. The ideal surgical technique remains debated, as various procedures aim for complete excision, tension-free closure, and low recurrence while ensuring early recovery and minimal morbidity [5–7]. Unfortunately, no single technique has proven superior due to variability in recurrence rates, healing time, and patient satisfaction [8].

Lay-open methods allow secondary healing but prolong recovery, while primary closure speeds healing yet carries higher risks of dehiscence and infection [9]. Novel flap-based reconstructions, such as the Limberg (LF) and keystone flaps (KSF), provide off-midline closure with robust vascularity, potentially reducing tension, infection, and recurrence [10–13].

The LF is a reliable rhomboid transposition flap but often requires longer operative time and wider dissection with subsequent seroma formation and tissue trauma [13, 14]. In contrast, the KSF preserves vascularity, minimizes tension on wound edges, and may enhance healing [15].

Although several studies compared these techniques regarding recurrence and healing, few have assessed their impact on functional recovery and patient satisfaction. This randomized comparative study evaluated the LF and KSF in PSD repair, hypothesizing that flap design significantly affects postoperative recovery and satisfaction, and aimed to identify factors independently predicting patient-reported outcomes.

## Methods

### Study design and ethical considerations

This 1-year prospective randomized comparative study was conducted at the surgery unit in a single surgical tertiary center. A sample size of 50 patients was determined on the basis of medium effect size (Cohen’s *d* = 0.6), 80% power, and alpha of 0.05, targeting patient satisfaction as the primary outcome. Prior to the initiation of the study, approval was obtained from the institutional ethics committee (institutional review board [IRB] NO: 0001289). Patient identities have been anonymized. The study was conducted following the principles of the Declaration of Helsinki, and all participants wrote an informed consent prior to the operation.

### Patients’ selection and randomization

A total of 62 patients with symptomatic PSD were assessed for eligibility. A total of 50 patients met the inclusion criteria and were randomized equally into 2 groups (*n* = 25 each):

### Group A (LF)

Patients underwent PSD excision followed by Limberg flap reconstruction.

### Group B (KSF)

Patients underwent PSD excision followed by keystone flap reconstruction.

Allocation and flap choice was concealed using sealed opaque envelopes opened immediately prior to the surgery.

**Fig. 1:**
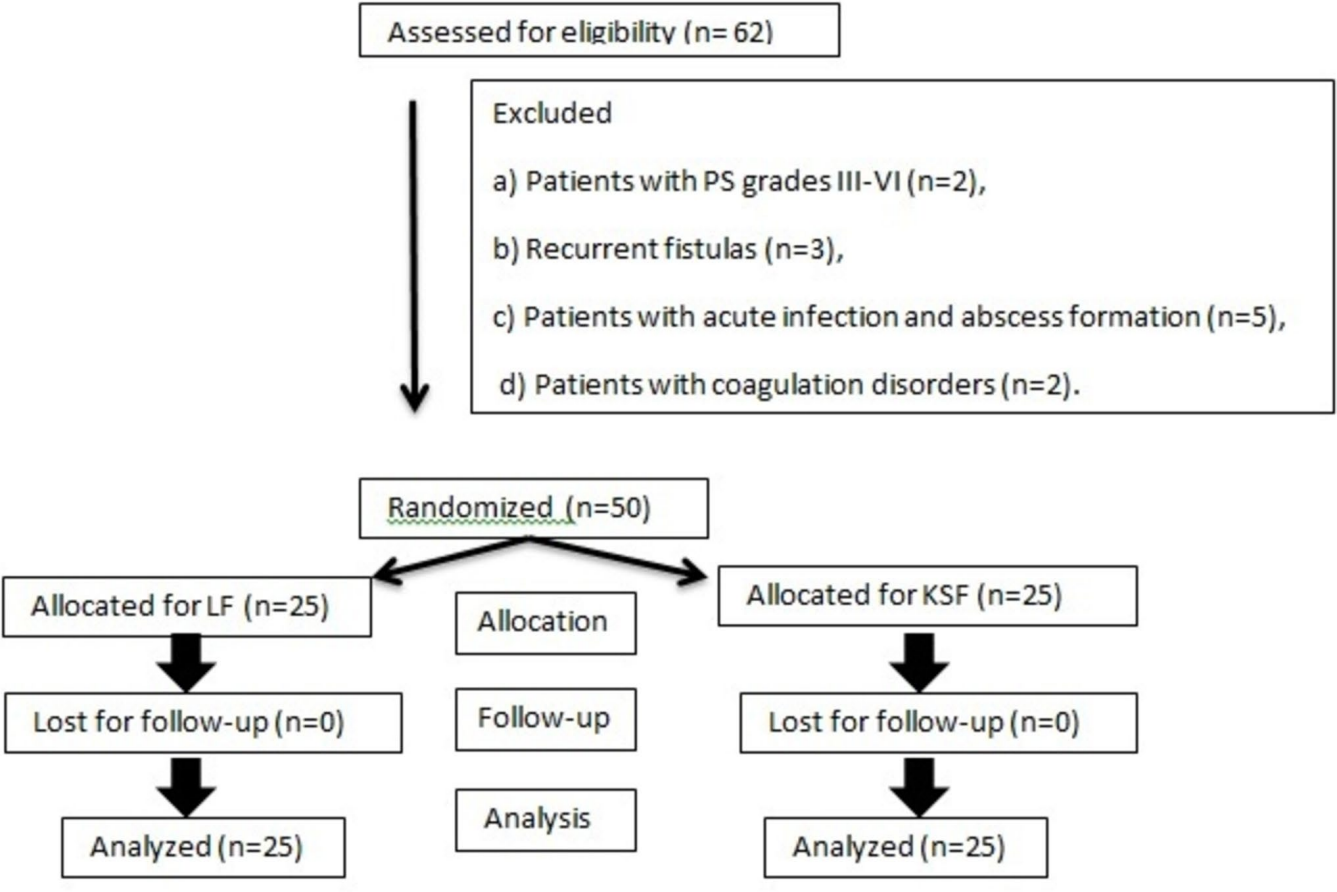
CONSORT flow diagram of patient enrollment, randomization, follow-up, and analysis

The inclusion criteria for the study encompassed all patients presenting with symptomatic simple pilonidal sinus (PS) grades I–II, requiring surgical excision with flap reconstruction, who will to adhere to a follow-up schedule. Exclusion criteria involved (1) patients with more complex PS grades III–VI (*n* = 2), (2) patients with recurrent fistulas (*n* = 3), (3) patients with acute infection and abscess formation (*n* = 5), (4) patients with coagulation disorders (*n* = 2). The Consolidated Standards of Reporting Trials (CONSORT) flow diagram detailing patient recruitment and allocation is shown in Fig. 1.

### Study outcomes

Patient satisfaction was the primary endpoint, assessed at 6 months postoperatively using a structured 5-point scale evaluating comfort, pain relief, healing experience, return to activity, and overall satisfaction with the surgical outcome (1 = least satisfied, 5 = most satisfied).

Secondary measures included key surgical and clinical parameters:

- Intraoperative parameters—operative time, blood loss, and flap reconstruction duration.
- Postoperative recovery parameters—hospital stay, wound-healing time, flap sensation, postoperative pain (Visual Analog Scale at 6, 12, and 24 h), and time to return to work or daily activity.
- Complications—seroma (localized serous collection), hematoma (blood collection requiring evacuation), wound infection (Centers for Disease Control and Prevention [CDC] criteria), wound dehiscence (partial: superficial layer separation; total: full-thickness wound opening), and flap necrosis (irreversible tissue loss requiring debridement or secondary repair).
- Recurrence rate—defined as the reappearance of pilonidal sinus symptoms within 6 months of surgery.

These parameters collectively determined the overall clinical performance and patient-centered benefit of the KSF and LF flap techniques.

### Procedure selection

All patients presenting with PS were initially counseled about the available surgical modalities for PSD. Only patients who consented to flap reconstruction were enrolled in the study. For these patients, the specific type of performed flap (KSF or LF) was determined strictly by random allocation using sealed opaque envelopes, opened immediately prior to surgery ensuring allocation concealment and avoiding surgeon preference bias.

#### Surgical technique

All procedures were performed under spinal anesthesia in the prone position by two experienced general surgeons with equal case distribution to minimize bias. Standard preoperative shaving, antiseptic preparation, and prophylactic antibiotics were applied in both groups.

**Fig. 2:**
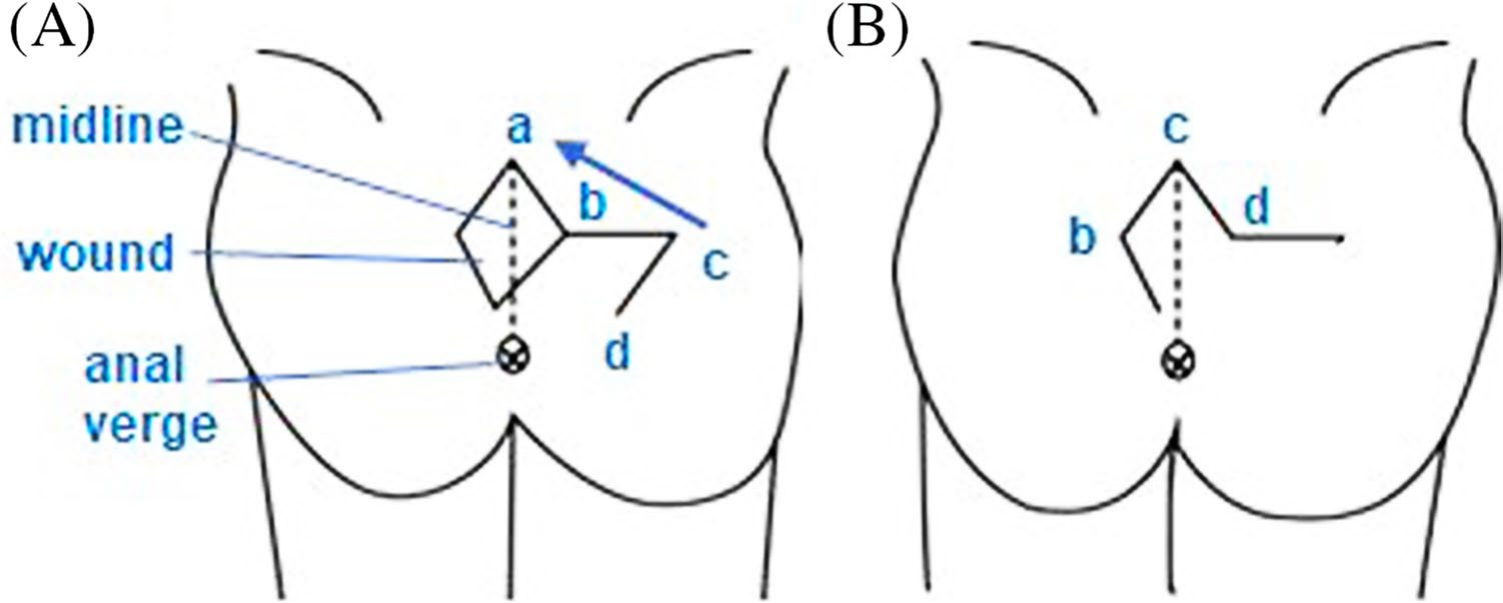
Limberg flap (diagrammatic design). **A** Rhomboid excision. **B** Limberg flap and construction after excision. Arrow direction points to the direction of flap transfer [13]

**Fig. 3:**
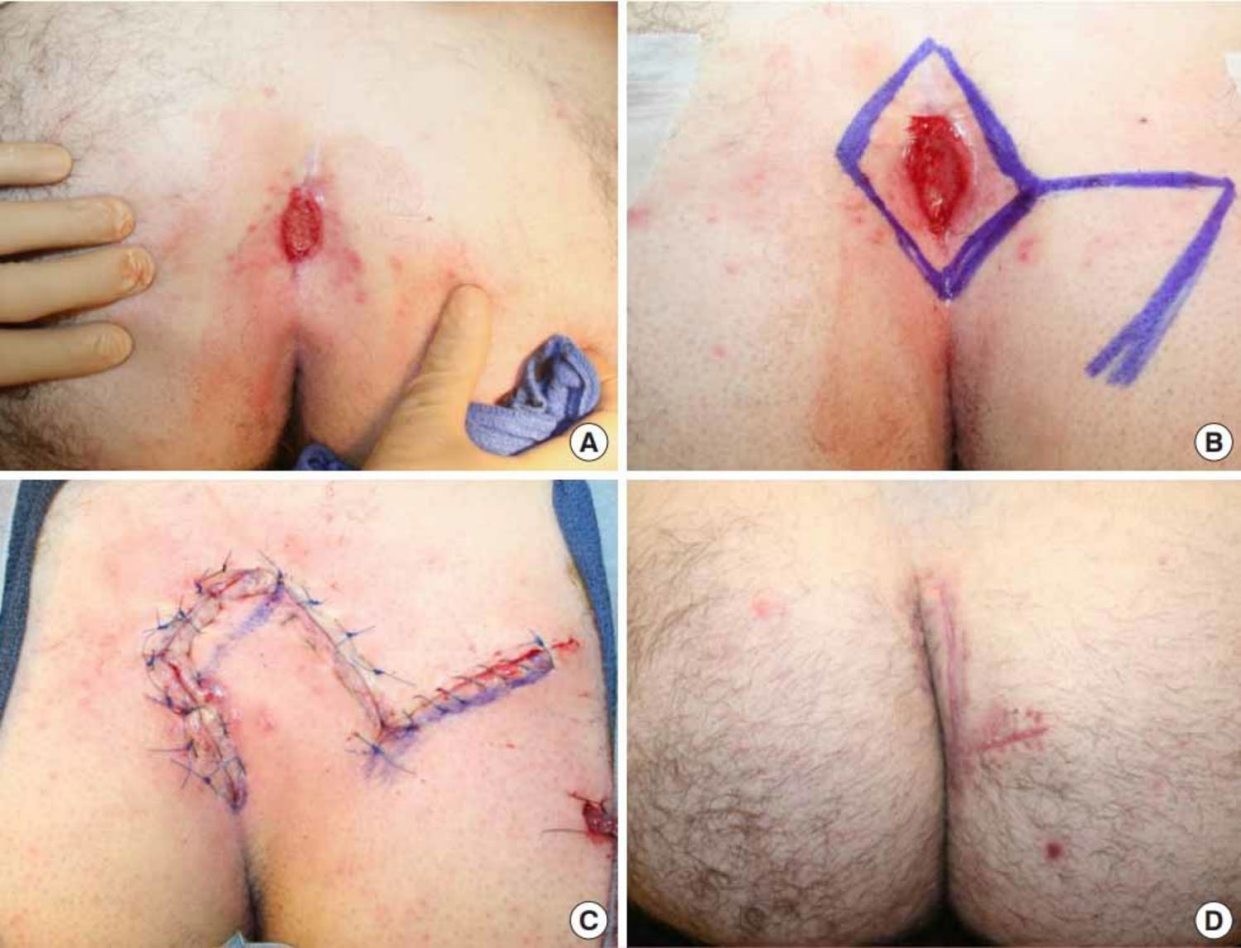
The Limberg flap (operative pictures). **A** Preoperative photograph. **B** Intraoperative markings. **C** Intraoperative photograph after excision and Limberg flap reconstruction. **D** Photograph taken 6 weeks postoperatively [17]

**Fig. 4:**
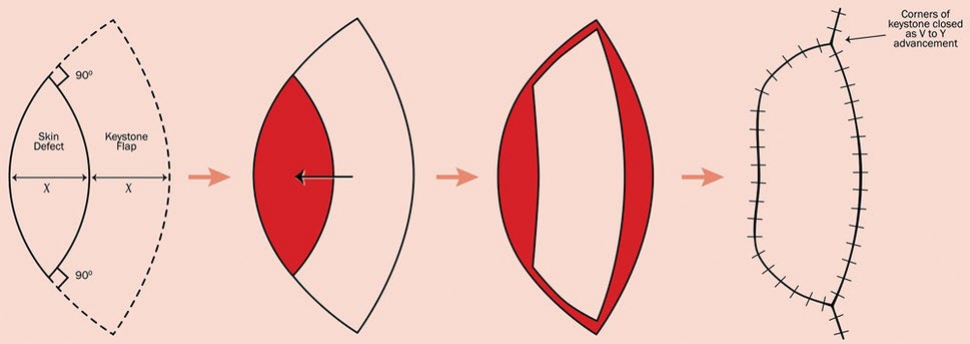
The KSF (diagrammatic design [19]

**Fig. 5:**
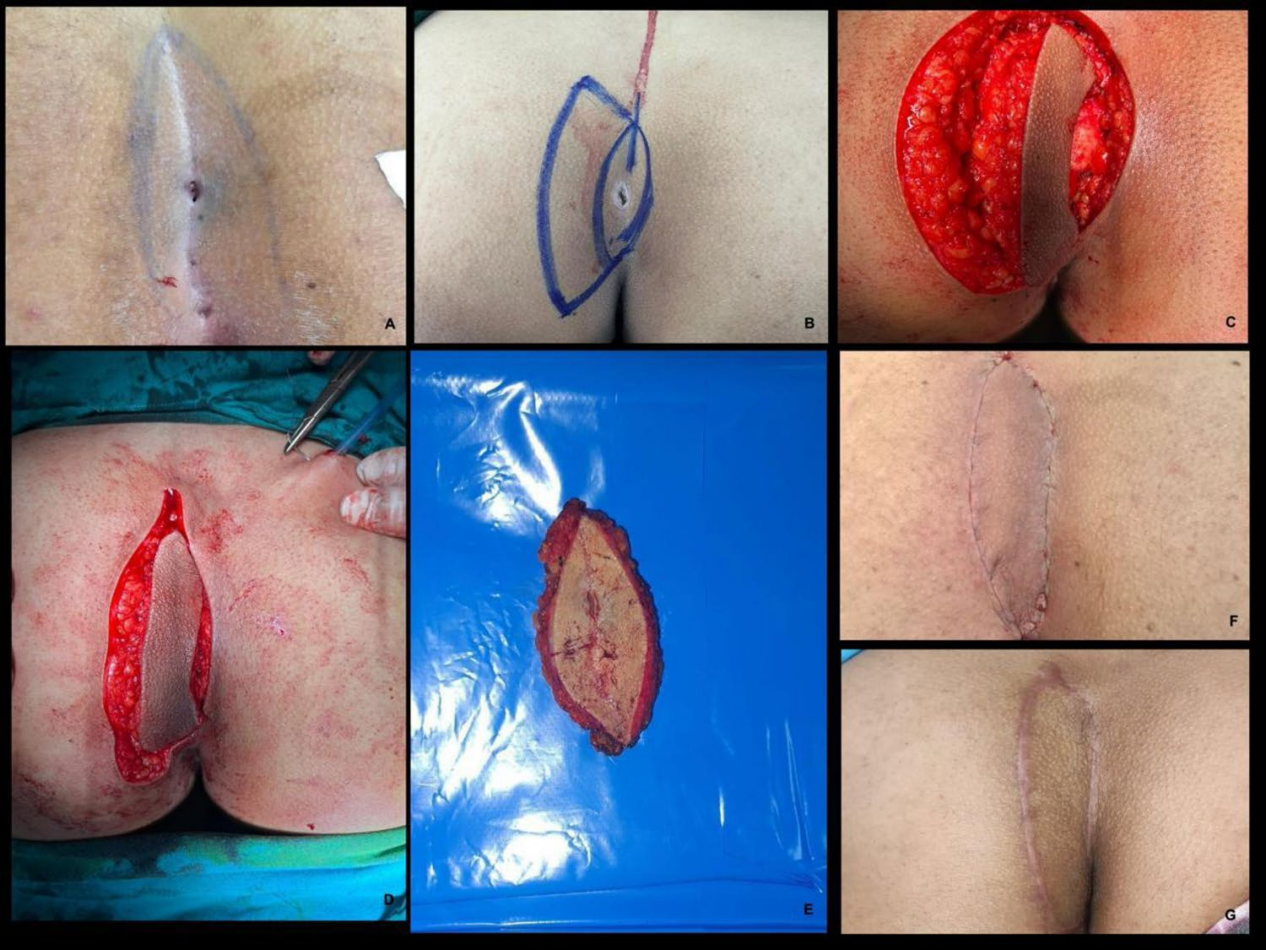
KSF technique (operative pictures from our cases). **A** Preoperative design. **B** Intraoperative PS resection with flap reconstruction. **C** Early postoperative view. **D** Late postoperative view

**Fig. 6:**
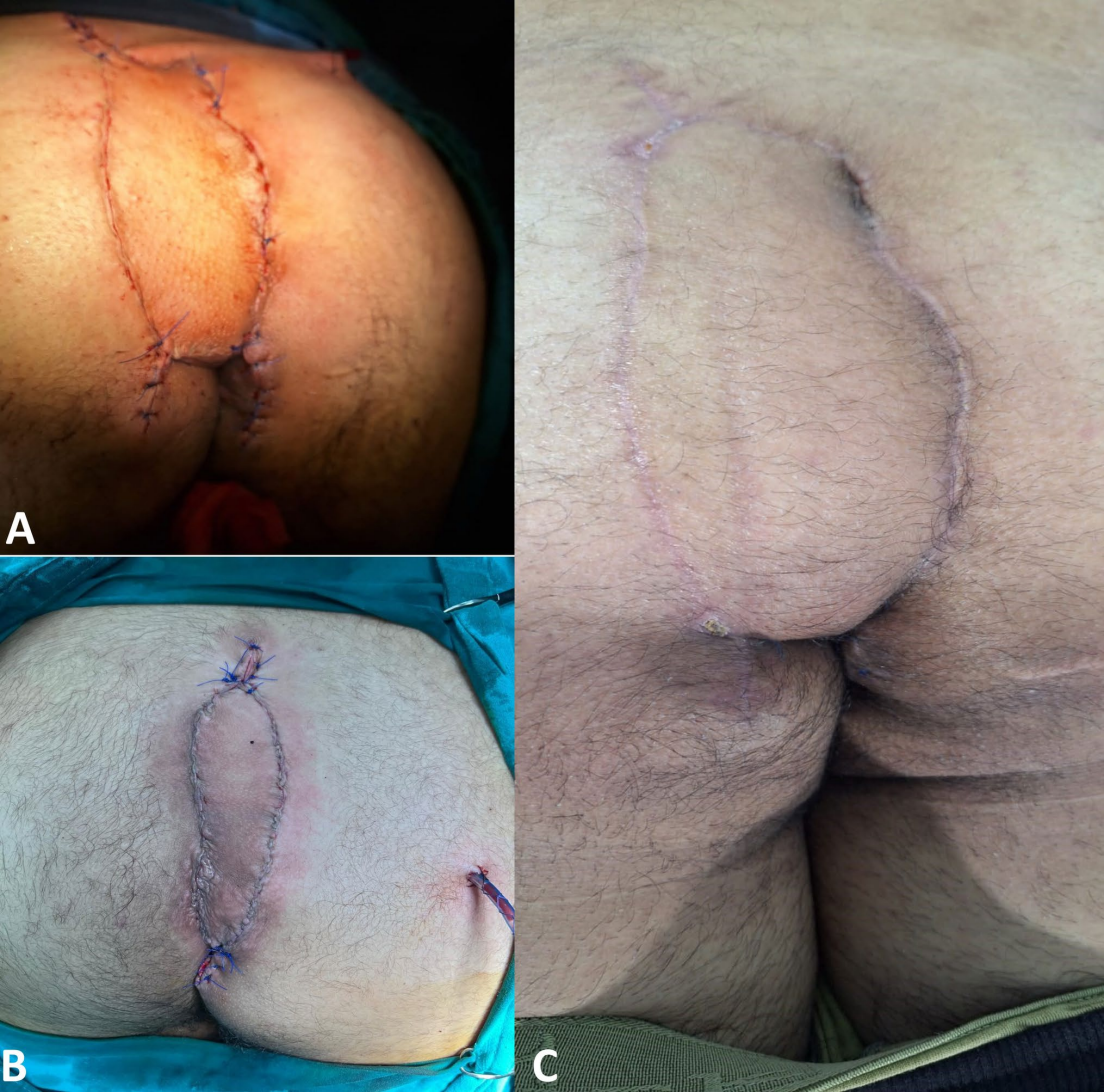
Different cases from our series where KSF was used to cover larger pilonidal sinus defects

- **The LF group (**Figs. 2, 3**)**: A rhomboid-shaped excision was performed to remove the sinus tract down to the presacral fascia (Fig. 2A). A fasciocutaneous transposition flap was then fashioned and rotated medially to fill the defect (Fig. 2B), following the technique described by Mentes et al. [16]. Hemostasis was achieved, and layered closure was performed [13, 17].
- **The KSF (**Figs. 4, 5, 6**):** A curvilinear trapezoidal excision of the sinus and affected tissues was carried out. A keystone perforator island flap with a 1:1 width-to-defect ratio was designed and advanced into the defect with minimal undermining to preserve perforators, as described by Behan [18, 19]. Closure was achieved without leaving dead space.

#### Postoperative care

All patients received standardized care, including daily dressings and a 1-week course of oral antibiotics. They were advised to lie prone or laterally for 48 h to reduce tension. Strenuous activity was restricted for 2 weeks. Follow-up visits were scheduled weekly for the first month and monthly thereafter for 1 year to monitor healing, complications, and recurrence. Flap sensation was assessed at 6 months using a bedside pin-prick test, recorded as either present or absent. This simple clinical method was chosen for its practicality in outpatient evaluation.

### Statistical analysis

Categorical variables were expressed as counts and percentages and analyzed using the chi-squared test or Fisher’s exact test. Continuous variables were tested for normality using the Shapiro–Wilk test. Normally distributed data were expressed as mean ± standard deviation (SD) and analyzed using Student’s *t*-test. Non-normally distributed data were expressed as median (interquartile range [IQR]) and analyzed using Mann–Whitney test. A *p*-value ≤ 0.05 was considered statistically significant. Data were analyzed using IBM SPSS v20.0.

## Results

This 1-year prospective study included 50 patients (40 male, 10 female) with simple PSD conducted at a tertiary referral center with a mean follow-up period of 1 year.

### Demographic and baseline characteristics

Between the specified dates, 50 consecutive PS patients (10 female, 40 male) underwent KSF or LF procedures. There was no statistically significant difference between the groups in terms of baseline characteristics, including body mass index (BMI), smoking, and diabetes, ensuring a balanced comparison (Table 1).

**Table 1:** Comparison between the two studied groups according to demographic data

	LF group (*n* = 25)			KSF group (*n* = 25)		Test of Sig.	*p*-Value
	No	Percentage (%)		No	Percentage (%)		
Sex							
Male	19	76.0		21	84.0	χ^2^ = 0.500	0.480
Female	6	24.0		4	16.0		
Age (years)							
Min–Max	19.0–49.0			21.0–49.0		*U* = 281.00	0.540
Mean ± SD	30.36 ± 7.66			29.76 ± 8.38			
Median (IQR)	29.0 (25.0–35.0)			27.0 (24.0–31.0)			
BMI (kg/m^2^)							
Min–Max	24.0–35.0			25.0–36.0		*t* = 0.255	0.800
Mean ± SD	28.68 ± 3.21			28.92 ± 3.45			
Median (IQR)	28.0 (26.0–31.0)			28.0 (26.0–32.0)			
DM							
No	21		84	22	88	χ^2^ = 0.166	1.000
Yes	4		16	3	12		
ASA score							
I	10		40.0	10	40.0		
II	11		44.0	9	36.0	0.600	0.741
III	4		16.0	6	24.0		
Smoking							
No	21		84	22	88	χ^2^ = 0.166	1.000
Yes	4		16	3	12		

The *p*-value column is the *p*-value for comparing between the two studied groups. *ASA* American Society of Anesthesiologists, *DM* diabetes mellitus, *Max* maximum, *Min* minimum, *Sig.* significance, *t* Student *t*-test, *U* Mann–Whitney *U* test

^*^Statistically significant at *p* ≤ 0.05

### History of the patients’ present illness

All patients presented with pilonidal sinus symptoms lasting 3–10 months. The mean duration was similar between groups (LF 6.5 ± 2.1 months versus KSF 6.4 ± 1.8 months, *p* = 0.777). Pain at the sacrococcygeal region was the most common complaint (24%), followed by purulent discharge (28% LF versus 20% KSF, *p* = 0.509). Most patients had multiple sinus openings (32% LF versus 26% KSF), with lateral pit involvement being more frequent than midline (68% LF versus 80% KSF, *p* = 0.333), while isolated midline pits were observed in 32% of LF cases and 20% of KSF cases.

### Intraoperative data (Table 2)

**Table 2:** Comparison between the two studied groups according to intraoperative data

	LF group (*n* = 25)		KSF group (*n* = 25)		Test of Sig.	*p*-Value
	No	Percentage (%)	No	Percentage (%)		
Type of anesthesia						
General	0	0.0	0	0.0	—	^—^
Spinal	25	100.0	25	100.0		
Defect size						
Defect length (cm)	5.9 ± 1		5.6 ± 0.8			0.09
Defect width (cm)	4.2 ± 0.4		3.7 ± 0.7			0.42
Total operative time						
Min–Max	61.0–86.0		50.0–65.0		*t* = 10.555^*^	< 0.001^*^
Mean ± SD	73.44 ± 7.94		54.96 ± 3.69			
Median (IQR)	73.0 (65.0–79.0)		55.0 (52.0–58.0)			
Resection time						
Min–Max	15.0–25.0		11.0–25.0		*U* = 232.50	0.108
Mean ± SD	19.80 ± 4.20		17.28 ± 2.97			
Median (IQR)	20.0 (15.0–25.0)		16.0 (15.0–20.0)			
Flap reconstruction time						
Min–Max	43.0–71.0		32.0–45.0		*t* = 9.054^*^	< 0.001^*^
Mean ± SD	53.64 ± 8.12		37.84 ± 3.20			
Median (IQR)	50.0 (48.0–59.0)		38.0 (35.0–40.0)			
Blood loss (mL)						
< 50	13	52.0	13	52.0	χ^2^ = 0.381	^MC^*p* = 1.000
50–100	10	40.0	11	44.0		
> 100	2	8.0	1	4.0		
Min–Max	20.0–130.0		20.0–120.0		*U* = 275.00	0.465
Mean ± SD	59.20 ± 31.35		52.80 ± 27.65			
Median (IQR)	45.0 (35.0–80.0)		45.0 (30.0–70.0)			

*Max* maximum, *MC* Monte Carlo test,*Min* minimum, *Sig.* significance, *t* Student *t*-test, *U* Mann–Whitney *U* test

The excised defect dimensions were comparable between groups. Mean defect length was 5.6 ± 0.8 cm in the KSF group and 5.9 ± 1.0 cm in the LF group (*p* = 0.09), while defect width was 4.2 ± 0.4 cm versus 3.7 ± 0.7 cm, respectively (*p* = 0.42).The KSF group showed a significantly shorter operative time (54.9 ± 3.7 min versus 73.4 ± 7.9 min, *p* < 0.001), mostly attributed to a faster flap reconstruction time (38.4 ± 3.2 min versus 53.6 ± 8.1 min, *p* < 0.001). Mean blood loss was lower with KSF (25.8 ± 2.8 mL versus 59.2 ± 3.1 mL, *p* < 0.001). No intraoperative complications, conversions, or transfusions occurred, confirming both techniques were safe regarding operative bleeding and technical feasibility.

### Postoperative pain and comfort (Table 3)

**Table 3:** Comparison between the different periods according to pain score

Pain score	2 h	6 h	12 h	24 h	Fr	*p*-Value
LF group (*n* = 25)						
Min–Max	6.0–10.0	4.0–7.0	1.0–4.0	0.0–1.0	74.711^*^	< 0.001^*^
Mean ± SD	8.48 ± 1.16	5.24 ± 0.83	2.28 ± 0.94	0.12 ± 0.33		
Median (IQR)	8.0 (8.0–9.0)	5.0 (5.0–6.0)	2.0 (2.0–3.0)	0.0 (0.0–0.0)		
*p*_0_		0.006^*^	< 0.001^*^	< 0.001^*^		
Sig. bet. periods		*p*_1_ = 0.005^*^, *p*_2_ < 0.001^*^, *p*_3_ = 0.009^*^				
KSF group (*n* = 25)						
Min–Max	6.0–10.0	2.0–4.0	1.0–2.0	0.0–1.0	73.866^*^	< 0.001^*^
Mean ± SD	8.44 ± 1.12	2.76 ± 0.72	1.28 ± 0.46	0.04 ± 0.20		
Median (IQR)	8.0 (8.0–9.0)	3.0 (2.0–3.0)	1.0 (1.0–2.0)	0.0 (0.0–0.0)		
*p*_0_		0.004^*^	< 0.001^*^	< 0.001^*^		
Sig. bet. periods		*p*_1_ = 0.014^*^, *p*_2_ < 0.001^*^, *p*_3_ = 0.005^*^				

Sig. bet. periods determined using Post Hoc Test (Dunn’s). *p* is the *p*-value for comparing between the two studied groups; *p*_0_ is the *p*-value for comparing between 2 h and each other period. *IQR* interquartile range, *Max* maximum, *Min* minimum, *SD* standard deviation, *Fr* Friedman test, *Sig. bet.* significance between

^*^Statistically significant at *p* ≤ 0.05

Postoperative pain scores were significantly lower in the KSF group at all early time points. At 6 h postoperatively, pain was 2.76 ± 0.72 in the KSF group versus 3.24 ± 0.83 in the LF group (*p* < 0.001). At 12 h, scores were 1.28 ± 0.46 versus 2.28 ± 0.94 (*p* < 0.001), while at 24 h, pain was minimal and comparable between groups (*p* = 0.302).

### Complications (Table 4)

**Table 4:** Comparison between the two studied groups according to incidence of postoperative complications

	LF group (*n* = 25)		KSF group (*n* = 25)		χ^2^	*p*-Value
	No	Percentage (%)	No	Percentage (%)		
Return to the operating room	0	0.0	0	0.0	—	—
Seroma formation	6	24.0	3	12.0	1.220	^FE^*p* = 0.463
Hematoma formation	0	0.0	0	0.0	—	—
Wound site infection	4	16.0	2	8.0	0.758	^FE^*p* = 0.667
Wound dehiscence						
No	21	84.0	23	92.0	1.091	^MC^*p* = 0.799
Yes, partial	3	12.0	1	4.0		
Yes, total	1	4.0	1	4.0		
Flap necrosis	0	0.0	0	0.0	—	—

*FE* Fisher exact test, *MC* Monte Carlo test

^*^Statistically significant at *p* ≤ 0.05

Postoperative complications were generally low in both groups, though trends favored the KSF flap. Seroma occurred in 24% of LF and 12% of KSF patients (*p* = 0.46), while wound infection was seen in 16% and 8%, respectively (*p* = 0.67). Partial wound dehiscence occurred in 12% of LF and 4% of KSF cases (*p* = 0.79). All complications were managed conservatively with daily dressings; no reoperations were required. Total dehiscence and flap necrosis were rare (one patient per group, *p* = 1.00). No major complications occurred, and both techniques were safe overall.

### Postoperative recovery and healing (Table 5)

**Table 5:** Comparison between the two groups according to postoperative recovery parameters

	LF group (*n* = 25)		KSF group (*n* = 25)		*U*	*p*-Value
Duration of drainage (days)						
Min–Max	6.0–9.0		5.0–7.0		*U* = 50.000^*^	< 0.001^*^
Mean ± SD	7.48 ± 0.82		5.80 ± 0.76			
Median (IQR)	7.0 (7.0–8.0)		6.0 (5.0–6.0)			
Wound healing time (days) = suture removal						
Min–Max	13.0–24.0		10.0–22.0		*U* = 117.00^*^	< 0.001^*^
Mean ± SD	17.08 ± 3.46		13.52 ± 3.15			
Median (IQR)	15.0 (14.0–20.0)		12.0 (12.0–15.0)			
Pain-free ambulation (days)						
Min–Max	7.0–10.0		5.0–9.0		*t* = 4.436^*^	< 0.001^*^
Mean ± SD	8.32 ± 0.95		6.96 ± 1.21			
Median (IQR)	8.0 (8.0–9.0)		7.0 (6.0–8.0)			
Return to normal activity (days)						
Min–Max	7.0–10.0		6.0–8.0		*U* = 50.00^*^	< 0.001^*^
Mean ± SD	8.48 ± 0.82		6.80 ± 0.76			
Median (IQR)	8.0 (8.0–9.0)		7.0 (6.0–7.0)			
Sensation of flap						
No	7	28.0	1	4.0	5.357^*^	0.049^*^
Yes	18	72.0	24	96.0		
Hospital stay (h)						
Min–Max	18.0–22.0		19.0–23.0			
Mean ± SD	20.06 ± 1.29		20.80 ± 1.32		2.000	0.051
Median (IQR)	20.0 (19.0–21.0)		21.0 (20.0–22.0)			

*IQR* interquartile range, *SD* standard deviation, *t* Student *t*-test, *U* Mann–Whitney *U* test

^*^Statistically significant at *p* ≤ 0.05

Postoperative recovery was significantly better in the KSF group, with faster wound healing and an earlier return to normal activity:

- Wound healing time was shorter in the KSF group (13.52 ± 3.15 days) compared with the LF group (17.08 ± 3.46 days, *p* < 0.001).
- The duration of wound drainage was also significantly shorter in the KSF group (5.80 ± 0.76 days versus 7.48 ± 0.82 days in the LF group, *p* < 0.001).
- Flap sensation was preserved in 96% of KSF patients compared with 72% in the LF group (*p* = 0.049).
- Pain-free ambulation was faster in the KSF group (6.96 ± 1.21 days versus 8.32 ± 0.95 days, *p* < 0.001). Patients in the KSF group returned to normal activity earlier (6.80 ± 0.76 days versus 8.48 ± 0.82 days in the LF group, *p* < 0.001).

### Patient satisfaction

Patient satisfaction, the primary outcome, was assessed 6 months postoperatively. Satisfaction was significantly higher in the KSF group, with 96% of patients reporting satisfaction versus 72% in the LF group (*p* = 0.046). Median satisfaction scores were 4.0 [IQR 4–5] for KSF and 2.0 [IQR 1–3] for LF (*p* < 0.001). Multivariate regression identified earlier return to normal activity (B = −0.739; 95% confidence interval [CI] −1.094 to −0.385; *p* < 0.001) and shorter wound-healing time (B = −0.137; 95% CI −0.237 to −0.038; *p* = 0.008) as independent predictors of satisfaction. Pain-free ambulation showed a borderline effect (*p* = 0.083), while operative time, drainage duration, and comorbidities were not significant (*p* > 0.05). These findings confirm that functional recovery parameters strongly influence patient satisfaction after flap surgery.

### Follow-up and recurrence in both groups

At 6 months postoperatively, recurrence was observed in only one patient (4%) in the LF group, while no recurrences were reported in the KSF group (*p* = 1.00). Although recurrence rates were low in both techniques, the absence of recurrence in the KSF group may indicate a potential long-term advantage (Tables 6, 7).

**Table 6:** Comparison between the two studied groups according to patient satisfaction

	LF group (*n* = 25)		KSF group (*n* = 25)		Test of Sig.	*p*-Value
	No	Percentage (%)	No	Percentage (%)		
Patient satisfaction						
1 (least satisfied)	7	28.0	1	4.0	χ^2^ = 27.018^*^	^MC^*p* < 0.001^*^
2	9	36.0	1	4.0		
3	6	24.0	2	8.0		
4	3	12.0	14	56.0		
5 (most satisfied)	0	0.0	7	28.0		
Min–Max	1.0–4.0		1.0–5.0		*U* = 68.000^*^	< 0.001^*^
Mean ± SD	2.20 ± 1.0		4.0 ± 0.96			
Median (IQR)	2.0 (1.0–3.0)		4.0 (4.0–5.0)			

*IQR* interquartile range, *SD* standard deviation, *U* Mann–Whitney *U* test, *MC* Monte Carlo test, *Sig.* significance

^*^Statistically significant at *p* ≤ 0.05

**Table 7:** Univariate and multivariate linear regression analysis for the parameters affecting patient satisfaction

	Univariate		^a^Multivariate	
	*p*-Value	B (LL–UL 95% CI)	*p*-Value	B (LL–UL 95% CI)
Total operative time	< 0.001^*^	−0.068 (−0.096 to −0.040)	0.258	−0.016 (−0.044 to 0.012)
Hospital stay	0.042^*^	0.285 (0.011–0.559)	0.970	0.003 (−0.134 to 0.139)
Duration of drainage (days)	< 0.001^*^	−0.675 (−0.945 to −0.405)	0.061	0.317 (−0.015 to 0.650)
Pain score (2 h)	0.873	0.027 (−0.314 to 0.369)		
Pain score (6 h)	< 0.001^*^	−0.481 (−0.703 to −0.259)	0.291	0.141 (−0.126 to 0.409)
Pain score (12 h)	< 0.001^*^	−0.723 (−1.104 to −0.343)	0.322	−0.119 (−0.361 to 0.122)
Pain score (24 h)	0.185	−0.924 (−2.305 to 0.457)		
Pain free ambulation (days)	< 0.001^*^	−0.732 (−0.948 to −0.516)	0.083	−0.158 (−0.337 to 0.022)
Return to normal activity (days)	< 0.001^*^	−0.873 (−1.090 to −0.656)	< 0.001^*^	−0.739 (−1.094 to −0.385)
Wound healing time (days)	< 0.001^*^	−0.280 (−0.343 to −0.216)	0.008^*^	−0.137 (−0.237 to −0.038)
Seroma formation	< 0.001^*^	−2.019 (−2.821 to −1.217)	0.670	0.274 (−1.022 to 1.570)
Wound site infection	< 0.001^*^	−2.197 (−3.184 to −1.210)	0.144	−1.673 (−3.948 to 0.601)
Wound dehiscence	< 0.001^*^	−1.188 (−1.768 to −0.607)	0.476	0.402 (−0.731 to 1.535)
Sensation of flap	< 0.001^*^	2.351 (1.565–3.138)	0.992	0.003 (−0.719 to 0.726)
Smoking	0.001^*^	−1.777 (−2.749 to −0.806)	0.768	−0.162 (−1.269 to 0.945)
DM	0.002^*^	−1.611 (−2.607 to −0.616)	0.464	0.419 (−0.729 to 1.566)
Limberg/keystone	< 0.001^*^	1.800 (1.243–2.357)	0.528	0.283 (−0.618 to 1.184)
BMI (kg/m^2^)	0.898	0.007 (−0.109 to 0.124)		

*B* unstandardized coefficients, *CI* confidence interval, *DM* diabetes mellitus, *LL* lower limit, *UL* upper limit

^a^All variables with *p* < 0.05 were included in the multivariate

^*^Statistically significant at *p* ≤ 0.05

## Discussion

This randomized study demonstrated that the KSF achieved faster recovery and higher patient satisfaction than the LF in PSD repair. Functional outcomes—particularly wound healing and return to normal activity—were the strongest independent predictors of satisfaction. To ensure homogeneity, the study included only patients with simple PSD; therefore, results apply primarily to such cases. Furthermore, the higher rate of lateral pits reflects disease progression but was evenly distributed, ensuring comparable baseline severity between groups. For extensive or recurrent sinuses, alternative methods such as the cleft-lift or Bascom technique may be more suitable.

The KSF showed a significantly shorter operative time (54.9 ± 3.7 min versus 73.4 ± 7.9 min, *p* < 0.001) despite comparable defect sizes, consistent with prior findings by Calisir and Ece [12], who reported shorter operative times for KSF. The perforator-based design of KSF likely reduces tissue mobilization, explaining its faster closure and minimal tension. This design may offer an advantage in patients with comorbidities such as diabetes or obesity. Although the LF group had slightly larger defects (5.9 × 4.2 cm versus 5.6 × 3.1 cm), the differences were not statistically significant (*p* = 0.09, 0.42), confirming balanced disease severity across groups.

As further evidence of the KSF’s versatility, our series included cases (Fig. 6) where relatively large midline defects were effectively closed using the KSF technique without tension or compromise to vascularity. This demonstrates that the KSF is not limited to small or moderate defects but can be safely applied in wider excisions when tissue laxity and vascular supply are adequate.

Although not statistically significant (*p* = 0.465), intraoperative blood loss was lower in the KSF group, with most patients losing < 100 mL. This may reflect the limited dissection required for KSF.

Postoperative complications were generally lower with KSF (6.6%) than with LF (12%), though not statistically significant. Rates of seroma, wound infection, and partial dehiscence all favored KSF. These trends align with prior studies by Roatis and Georgescu [15], who also reported fewer complications with the KSF. In this study, patients who developed complications had lower satisfaction scores, reflecting the functional and psychological burden of even minor postoperative morbidity.

Postoperative recovery was faster with KSF, demonstrated by lower pain scores, shorter hospital stay, and earlier wound healing and activity resumption—all key contributors to higher patient satisfaction.

Patients in the KSF group reported significantly lower postoperative pain scores at 6 and 12 h compared with the LF group, with no significant difference at 24 h. These results are likely due to less tissue tension and improved wound closure design. This finding is supported by Ekici et al. (2019) [20], who found that patients undergoing KSF experienced less early postoperative pain than those treated with LF. In our study, pain at 12 h had a strong negative correlation with satisfaction (*r* = −0.723, *p* < 0.001). Reduced early pain may enhance mobilization, decrease analgesic needs, and improve overall comfort.

The KSF group had a shorter mean hospital stay. though the difference was not statistically significant; hospital stay moderately correlated with satisfaction in univariate analysis (*p* = 0.042) but lost significance after adjustment, suggesting its effect is mediated through broader recovery parameters such as wound healing and return to activity.

Wound healing time was significantly shorter in the KSF group compared with the LF group. These findings align with Gulcu and Ozturk [21], who demonstrated that early recovery correlates with improved patient-reported outcomes. In multivariate regression, wound healing emerged as an independent predictor of patient satisfaction (B = −0.137, 95% CI −0.237 to −0.038; *p* = 0.008).

Earlier pain-free ambulation enhances patient autonomy, reduces the risk of venous thromboembolism, so contributing to higher patient satisfaction. The KSF patients achieved pain-free ambulation significantly earlier than those in the LF group, consistent with Milone et al. [22], who reported that minimally invasive techniques facilitate quicker recovery. Although not retained as an independent predictor in multivariate regression (*p* = 0.083), pain-free ambulation showed a strong negative association with satisfaction in univariate analysis (B = −0.732, *p* < 0.001), indicating that quicker recovery of mobility is an important determinant of perceived success.

The KSF group returned to normal activity significantly earlier than the LF group (B = −0.739, 95% CI −1.094 to −0.385; *p* < 0.001), which was the strongest independent determinant of patient satisfaction, as also observed by Calisir and Ece [12], although their study did not correlate return to activity with patient-reported outcomes. Only one recurrence occurred in the LF group. Despite the small sample, recurrence was clinically relevant and associated with the lowest satisfaction scores, as it undermines confidence in surgical success.

Flap sensation was preserved in 96% of KSF cases versus 72% of LF cases (*p* = 0.0449). This advantage likely stems from the perforator-based design of the KSF maintaining cutaneous nerve integrity with less tissue mobilization, unlike the broader dissection required in the LF technique. Although sensation correlated with satisfaction in univariate analysis (*p* = 0.001), it lost significance after adjustment (*p* = 0.768), probably due to overlap with other recovery variables. While our bedside pinprick test was a practical tool, future studies using objective sensory mapping are warranted.

Multivariate analysis revealed that only return to normal activity (*p* < 0.001) and wound-healing time (*p* = 0.008) independently predicted patient satisfaction, underscoring functional recovery as the principal determinant. Although favorable outcomes in the KSF group—such as less blood loss, faster ambulation, fewer complications, and better flap sensation—were significant in univariate tests, they lost significance after adjustment, indicating their effects were mediated through enhanced recovery. Flap type, while significant in univariate testing (*p* < 0.001), was not an independent predictor (*p* = 0.528). Thus, the choice of flap may influence recovery speed but not satisfaction directly. Overall, these findings reinforce the clinical value of the KSF, including minimal tension, preserved blood supply, and faster recovery; this translates into better satisfaction even when flap type itself is not a direct predictor after adjustment.

Limitations of this study includes the single-center design, small sample size, and 6-month follow-up duration. These factors may limit generalizability and the ability to detect long-term differences in recurrence or durability. Nonetheless, the randomized design and standardized technique strengthen the reliability of the findings. Larger multicenter trials with extended follow-up are warranted to confirm its long-term efficacy.

## Conclusion

The KSF demonstrated superior surgical outcomes, faster recovery, and higher patient satisfaction compared with the LF. Although multiple variables were evaluated, only wound-healing time and return to normal activity independently predicted satisfaction. These findings highlight that enhanced functional recovery, rather than flap design alone, plays the primary role in patient-reported success. Given the single-center design and limited sample size, larger multicenter studies are warranted to validate these results and evaluate long-term outcomes.

## Data Availability

No datasets were generated or analyzed during the current study.
